# Risk Factors Associated with Systemic Arterial Hypertension in Postmenopausal Women Engaged in Resistance Training: A Cross-Sectional Observational Study

**DOI:** 10.3390/ijerph23030408

**Published:** 2026-03-23

**Authors:** Renata Corrêa Arruda, Pablo Augusto Garcia Agostinho, Ítalo Santiago Alves Viana, Maria Luíza da Cruz Santos, Marcela Siqueira Benjamim, Paula Janyn Melo-Buitrago, Alice Ribeiro Cutis Vaz, Cláudia Eliza Patrocínio de Oliveira, Édison Andrés Pérez-Bedoya, Osvaldo Costa Moreira

**Affiliations:** 1 Department of Medicine and Nursing, Federal University of Viçosa, Viçosa Campus, Viçosa 36570-900, MG, Brazil; renata.arruda@ufv.br; 2 Human Morphophysiology Analysis Laboratory (HUMAN), Federal University of Viçosa, Viçosa Campus, Viçosa 36570-900, MG, Brazil; pablo.agostinho@ufv.br (P.A.G.A.); italo.viana@ufv.br (Í.S.A.V.); maria.l.cruz@ufv.br (M.L.d.C.S.); marcela.benjamim@ufv.br (M.S.B.); paula.janyn@ufv.br (P.J.M.-B.); alice.r.vaz@ufv.br (A.R.C.V.); cpatrocinio@ufv.br (C.E.P.d.O.); 3 Department of Physical Education, Federal University of Viçosa, Viçosa Campus, Viçosa 36570-900, MG, Brazil; 4 Faculty of Physical Education, Recreation, and Sport, Jaime Isaza Cadavid Colombian Polytechnic, Medellín 50001, Colombia; eaperez@elpoli.edu.co

**Keywords:** menopause, strength training, aging

## Abstract

**Highlights:**

**Public health relevance—How does this work relate to a public health issue?**

- Systemic arterial hypertension (SAH) remains highly prevalent among postmenopausal women, even in those engaged in structured resistance training programs.
- Central adiposity and lower educational level were identified as key factors associated with SAH in physically active older women, highlighting persistent cardiovascular vulnerability in this population.

**Public health significance—Why is this work of significance to public health?**

- This study addresses a gap in epidemiological evidence by focusing on physically active postmenopausal women, a group often underrepresented in public health analyses.
- Findings reinforce that physical activity level alone may not fully mitigate cardiometabolic risk when excess central adiposity and socioeconomic determinants are present.

**Public health implications—What are the key implications or messages for practitioners, policy makers and/or researchers in public health?**

- Public health strategies targeting postmenopausal women should integrate resistance training with interventions aimed at reducing central adiposity and addressing social determinants such as educational inequality.
- Multidisciplinary and preventive approaches are essential to monitor and manage cardiovascular risk factors in aging women, even among those classified as physically active.

**Abstract:**

Background: Systemic arterial hypertension (SAH) shows a high prevalence among postmenopausal women and represents an important public health concern. Objective: To evaluate factors associated with SAH in postmenopausal women participating in a resistance training program. Methods: This observational, cross-sectional study included 55 postmenopausal women (66.0 ± 4.9 years) recruited from the “More Active Women” research project, an umbrella experimental and longitudinal study involving resistance training interventions. Cross-sectional data were collected during the baseline assessment (April–May 2025). Sociodemographic variables, nutritional status (body mass index and waist circumference), and behavioral and health-related variables obtained through structured interviews and anthropometric assessments were analyzed. Associations were tested using Pearson’s chi-square test or Fisher’s exact test, with effect size estimated by Phi or Cramer’s V when appropriate, and binary logistic regression was performed for adjusted analyses. Results: Significant associations were observed between SAH and elevated BMI (*p* = 0.03; φ = 0.30), waist circumference > 88 cm (*p* = 0.006; φ = 0.40), and lower educational level (*p* = 0.003; V = 0.47). In the adjusted analysis, waist circumference ≤ 88 cm was associated with a lower likelihood of SAH (OR = 5.54; 95% CI: 0.965–31.872; *p* = 0.007), whereas lower educational level was associated with a higher likelihood of hypertension (OR = 13.98; 95% CI: 1.505–129.833; *p* = 0.004). Conclusion: Excess central adiposity and lower educational level are associated with SAH in postmenopausal women, highlighting the importance of integrated health promotion strategies that address both cardiometabolic risk factors and social determinants of health during aging.

## 1. Introduction

Systemic arterial hypertension (SAH) is one of the leading causes of cardiovascular morbidity and mortality worldwide, constituting an important modifiable risk factor for adverse events such as stroke, myocardial infarction, and heart failure [1]. Although the prevalence of SAH among young adults is higher in men, a marked increase in SAH is observed in women after menopause, mainly due to the hormonal and metabolic changes characteristic of this stage of life; nevertheless, its prevalence remains lower than that observed in men, even after menopause [2].

The menopausal transition is primarily characterized by a reduction in estrogen levels, which compromises vasodilatory mechanisms and the modulation of endothelial function, lipid metabolism, and insulin sensitivity [3]. These changes favor increased arterial stiffness, central fat accumulation, and elevated blood pressure, factors directly associated with a higher risk of SAH and other cardiovascular outcomes in postmenopausal women [4].

In this context, the regular practice of physical activity stands out as a non-pharmacological intervention widely promoted and validated worldwide as one of the most effective strategies for the prevention and control of SAH, particularly in populations with a cardiovascular vulnerability profile such as postmenopausal women [5].

Evidence from systematic reviews of randomized clinical trials indicates that aerobic or combined exercise programs (aerobic plus resistance) promote significant reductions in systolic and diastolic blood pressure in postmenopausal women, in addition to improvements in lipid profile and cardiorespiratory fitness [6,7]. However, studies investigating factors associated with SAH specifically in physically active postmenopausal women engaged in resistance exercise remain limited. This population is often underrepresented in epidemiological analyses, which tend to include predominantly sedentary individuals or mixed samples.

Therefore, gaps persist in the understanding of sociodemographic, behavioral, and health-related determinants associated with SAH in this specific group. Thus, the objective of the present study was to evaluate factors associated with SAH in postmenopausal women practicing resistance exercise.

## 2. Materials and Methods

### 2.1. Study Design

This is an observational, cross-sectional, and descriptive study conducted using a convenience sampling approach within the research project “Mulheres Mais Ativas”, an umbrella study of an experimental and longitudinal nature in which two groups of women underwent resistance training (inertial flywheel versus traditional resistance training) for nine months, with the primary objective of evaluating the behavior of strength-related outcomes. The cross-sectional data used in the present analysis were collected at baseline between April and May 2025. The project was approved by the Human Research Ethics Committee of the Federal University of Viçosa (UFV) (approval number 1.821.139) and registered at ClinicalTrials.gov (NCT06758206).

Participants were recruited through announcements disseminated by the Municipal Health Department of Viçosa, Minas Gerais, Brazil, as well as through communication channels of UFV (radio and email). After this initial stage, the Informed Consent Form was provided for reading and signature, with one copy delivered to the participant and another retained by the research team coordinator.

**Figure 1:**
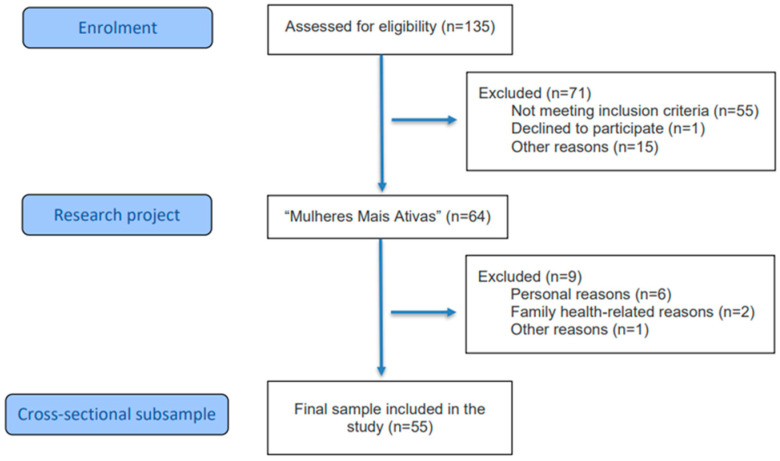
Flowchart of study participants.

The sample was defined by convenience, considering the total number of eligible participants from the umbrella project “Mulheres Mais Ativas” who met the inclusion criteria during the data collection period. Initially, 135 women were assessed for eligibility. Of these, 71 were excluded for refusing to participate and/or for not meeting the eligibility criteria: age ≥ 60 years; medical clearance to perform resistance exercise; at least 24 months of amenorrhea; and a minimum score of 24 points on the Mini-Mental State Examination (MMSE) [8]. The exclusion criteria were: participation in other resistance training programs within the three months preceding the training protocol and during the data collection period; and diagnosis of untreated joint, chronic, or psychiatric diseases. Thus, 64 women were eligible at the beginning of the study. However, nine women withdrew from participation due to personal reasons or family health issues, resulting in a final sample of 55 women (see Figure 1). The study was conducted at the Human Morphophysiology Laboratory (HUMANLAB) of the Federal University of Viçosa.

### 2.2. Resistance Training Program

The resistance training program was supervised by physical education professionals and undergraduate students previously trained for the study procedures. Training sessions were conducted individually or in small groups of up to three women per instructor to ensure adequate supervision and proper exercise execution. Both groups performed six standardized exercises targeting major muscle groups: knee extension, knee flexion, plantar flexion, scapular retraction, elbow flexion, and shoulder abduction.

The training program started in March and ended in December, lasting nine months. Sessions were performed twice per week on nonconsecutive days and lasted approximately 50 min, including warm-up, exercise execution, and rest intervals. Attendance was monitored throughout the intervention to ensure adherence to the training protocol.

The inertial flywheel resistance training group used a multi-leg iso-inertial device and the T-one iso-inertial platform (Physical Solutions, São Paulo, Brazil). Participants performed four sets of eight coupled concentric and eccentric actions at high intensity (score of 10 on the OMNI-RES scale), with 120 s of rest between sets. Load progression was self-regulated according to the velocity used to mobilize the flywheel (constant inertial load of 0.050 kg/m^2^). Exercises were performed with maximal concentric power, followed by braking during the final third of the eccentric phase, without pause between the concentric and eccentric actions. The same verbal encouragement and eccentric braking technique described by [9] were adopted.

The traditional resistance training group used weight machines and free weights available in the strength training laboratory. Four sets of eight to twelve repetitions were prescribed at moderate to high intensity (scores between 6 and 10 on the OMNI-RES scale), with 60 s of rest between sets. Load progression occurred when participants were able to perform more than 12 repetitions with proper technique or reported a perceived exertion below 6 on the OMNI-RES scale. In such cases, the external load was progressively increased by 2–5% of the weight lifted. Participants were instructed to perform each repetition with a one-second concentric action, no pause between phases, and a two-second eccentric action.

### 2.3. Sociodemographic, Nutritional Status, and Behavioral Information

Variables considered risk factors for systemic arterial hypertension (SAH) were analyzed, including sociodemographic characteristics (age, race/skin color, and educational level: Elementary School I, 0–7 years; Elementary School II, 8–11 years; and High School or Higher Education, ≥12 years), nutritional status assessed by body mass index (BMI) and waist circumference (WC), as well as behavioral and health-related variables such as physical activity level (PAL), smoking, alcohol consumption, and previous diagnosis of diabetes mellitus (DM).

Information was obtained through medical history and anthropometric assessment. During the medical history interview, participants were asked about previous use of hormone replacement therapy (HRT) and the time since discontinuation when applicable. Body weight was measured using a digital scale with bioelectrical impedance (Techline^®^, model TEC-180, São Paulo, SP, Brazil), with participants barefoot, wearing light clothing, and in an upright position. Height was measured using a portable vertical stadiometer (Sanny^®^, model ES2060—Personal Caprice, São Paulo, SP, Brazil), following the Frankfurt plane. BMI was calculated as body weight divided by height squared (kg/m^2^). Nutritional status was classified according to the criteria proposed by Lipschitz [10] for older adults, which consider BMI < 23 kg/m^2^ as underweight, 23–27.9 kg/m^2^ as normal weight, and ≥28 kg/m^2^ as overweight.

Waist circumference was measured using a non-elastic anthropometric tape (Teklife^®^, 150 cm, Tek-life, Beijing, China), positioned at the midpoint between the last rib and the iliac crest, with the measurement taken at the end of expiration. Classification followed the recommendations of the Brazilian Association for the Study of Obesity [11], with values of 80–88 cm indicating increased coronary risk and values ≥ 88 cm indicating very high coronary risk. Although recent evidence suggests that optimal waist circumference cut-off points may vary according to ethnicity, the threshold of ≥88 cm remains widely adopted in epidemiological studies and international cardiometabolic risk guidelines, allowing comparability with previous investigations. Considering the relatively small sample size of the present study, performing sensitivity analyses using multiple alternative cut-off points could result in very small subgroups and unstable statistical estimates. Continuous quantitative variables were categorized based on cutoff points from national and international guidelines.

PAL was initially classified into three categories (inactive: <150 min/week; active: 150–300 min/week or ≥600 METs/min/week; and very active: >300 min/week or ≥1500–3000 METs/min/week), according guideline [12], considering the total weekly volume of physical activity (supervised resistance training program + self-reported physical activities performed outside the program). However, for the bivariate analyses, PAL was reclassified into two categories in order to improve analytical stability and more accurately represent habitual physical activity performed outside the supervised resistance training program. Specifically, minutes corresponding to the resistance training sessions conducted as part of the research program were excluded from this secondary classification, since all participants were involved in a structured training protocol, which could artificially inflate the overall PAL. After this reclassification, participants were categorized as inactive (including those initially classified as inactive or active after excluding intervention-related activity) and active (those classified as very active based on habitual physical activity performed outside the program). This approach was adopted to minimize potential classification bias resulting from the structured intervention and to ensure a more appropriate distribution of frequencies in the statistical analyses.

Participants were classified regarding the presence or absence of systemic arterial hypertension (SAH) and DM based on self-reported previous diagnosis, confirmed by the chronic use of oral and/or injectable antihypertensive and/or antidiabetic medications prescribed by a licensed physician. After this stage, the sample was categorized into two groups according to SAH diagnosis: Group 1—SAH present and Group 2—SAH absent, for subsequent statistical analyses.

Potential selection biases resulting from convenience sampling and information biases related to the use of self-reported data for PAL, alcohol consumption, and smoking were considered. To minimize these biases, standardized protocols and trained professionals were used in the application of medical history interviews and anthropometric data collection, in addition to confirmation of the diagnosis of chronic health conditions (SAH and/or DM) through a copy attached to the medical history of the medical prescription for the chronic use of medication indicated for such conditions issued by a licensed professional.

### 2.4. Statistical Analysis

Statistical analyses were performed using the Statistical Package for the Social Sciences (SPSS), version 23.0. Categorical variables were described using absolute and relative frequencies, whereas quantitative variables were presented as mean and standard deviation (SD). The normality of quantitative variables was assessed using the Kolmogorov–Smirnov test.

Associations between categorical variables were analyzed using Pearson’s chi-square test or, when assumptions were not met, Fisher’s exact test. The effect size of the associations was evaluated using the Phi coefficient (φ) for 2 × 2 contingency tables and Cramér’s V for tables larger than 2 × 2. For multivariate analysis, binary logistic regression was performed to estimate odds ratios (OR) and their respective 95% confidence intervals (95% CI).

Sensitivity analyses were not performed due to the reduced sample size and the exploratory nature of the study. A significance level of 5% (*p* < 0.05) was adopted for all analyses. No missing data were identified for the variables included in the statistical analyses.

## 3. Results

The sample consisted of 55 postmenopausal women, as shown in Figure 1, with a mean age of 66.0 ± 4.9 years and a mean postmenopausal duration of 15.2 ± 5.1 years. Regarding HRT, 82% of the participants reported never having used this therapy. Among those who reported previous use, discontinuation occurred at least five years prior to study participation. Therefore, exposure to HRT was considered low in this sample and unlikely to substantially influence the cardiometabolic outcomes evaluated. Most participants self-identified as White (56.4%) and had an educational level of 12 years or more (67.3%). No missing data were observed for any of the analyzed variables. Regarding health behaviors, most women were classified as physically active (50.9%), while 11 were considered very active (20.0%) and 16 physically inactive (29.1%). Recreational alcohol consumption was reported by 28 women (51.0%), and the prevalence of smoking was low, observed in eight women (14.5%).

Health-related variables indicated a mean body weight of 68.8 ± 12.5 kg, a mean height of 1.56 ± 0.06 m, and a mean BMI of 27.95 ± 4.93 kg/m^2^. A high prevalence of women classified as overweight and/or obese (49.1%) and with WC ≥ 88 cm (70.9%) was observed, indicating a very high coronary risk for the development of metabolic complications in the studied sample. Additionally, 24 participants (43.6%) had a diagnosis of SAH and six (11.0%) had DM (see Table 1).

Results of the bivariate analysis showed a statistically significant association (*p* = 0.03) between BMI and SAH, with a higher proportion of hypertensive participants in the overweight + obesity group compared with the normal weight + underweight group, with a moderate effect size. Similarly, an association was observed between WC and SAH (*p* = 0.006), with a higher prevalence of hypertensive participants among those with WC greater than 88 cm, presenting a moderate to large effect size.

An association between SAH and educational level was also observed using Fisher’s exact test (*p* = 0.003), with a moderate to large effect size, indicating a higher prevalence of hypertension among participants with lower educational attainment (<12 years).

In contrast, no associations were observed between SAH and PAL (*p* = 0.73), SAH and smoking status (*p* = 0.71), SAH and alcohol consumption (*p* = 0.28), or SAH and race/skin color (*p* = 0.79). However, the variable DM showed a tendency toward association with SAH, with a small to moderate effect size, indicating a higher frequency of hypertension among participants with diabetes, although this association did not reach statistical significance (*p* = 0.07) (Table 2).

Binary logistic regression analysis showed that waist circumference and educational level remained independently associated with SAH. Participants with a waist circumference of 80–88 cm had lower odds of being diagnosed with SAH compared with those with values > 88 cm (crude OR = 0.11; 95% CI: 0.022–0.553; *p* = 0.007). Similarly, lower educational level (0–7 years) was associated with higher odds of SAH, with a thirteenfold greater likelihood of SAH among participants with lower educational attainment compared with those with higher educational level (≥12 years) (adjusted OR = 13.98; 95% CI: 1.505–129.833; *p* = 0.020; crude OR = 23.63; 95% CI: 2.690–207.667; *p* = 0.004), as presented in Table 3.

## 4. Discussion

The present study aimed to evaluate factors associated with SAH in postmenopausal women practicing resistance exercise. The main findings indicate that postmenopausal women, even when physically active, may present excess central adiposity measured by WC and high levels of overweight and obesity, and that this condition may be closely related to socioeconomic factors, such as educational level, and to pre-existing comorbidities, such as a previous diagnosis of DM. Therefore, there is a diversity of factors that may aggravate the risk of SAH in women undergoing the aging process, even among those who are physically active.

One of the risk factors associated with SAH in the studied sample relates to the nutritional status of the participants, particularly BMI and WC, both of which are widely recognized indicators of overall and central adiposity. Elevated values of these anthropometric markers are strongly associated with increased cardiometabolic risk and may contribute to the development and progression of hypertension through mechanisms such as insulin resistance, chronic low-grade inflammation, and activation of the renin–angiotensin–aldosterone system [13]. In postmenopausal women, these alterations are often intensified by hormonal changes, especially estrogen deficiency, which promotes the redistribution of body fat toward the abdominal region and favors visceral adiposity, as described by Lizcano and Guzmán [14]. This shift in fat distribution is associated with adverse metabolic profiles and increased cardiovascular risk, as described by Lovejoy et al. [15].

HRT has also been described as an important factor influencing cardiovascular risk in postmenopausal women, as it may attenuate some of the metabolic and vascular changes associated with estrogen deficiency, including alterations in lipid profile, endothelial function, and arterial stiffness [16]. In the present study, however, the influence of HRT was likely minimal, as the vast majority of participants (82%) reported never having used hormone therapy. Among those who had previously used HRT, treatment had been discontinued for at least five years prior to participation. This characteristic of the sample suggests that the cardiometabolic profile observed in this study is more strongly related to long-term postmenopausal physiological changes rather than to the potential modulatory effects of hormone therapy.

The tendency toward association observed between DM and SAH in the present sample is consistent with the well-established coexistence of these diagnoses, often described as part of a condition known as Metabolic Syndrome [17]. The lack of persistence of this association after adjustment in our sample may be explained by the small number of participants diagnosed with DM, which may have limited the stability of the estimates.

Regarding sociodemographic variables, an association was observed between SAH and participants with lower educational levels. This finding aligns with extensive national and international literature identifying educational attainment as an important health determinant, directly influencing the risk of cardiovascular diseases [18,19]. Lower educational levels are directly associated with reduced access to health information and greater exposure to risk factors, as well as lower adherence to preventive and therapeutic strategies, such as the use of HRT, as reported by Ferreira-Campos et al. [20] in a longitudinal study involving 1492 postmenopausal women.

Although PAL did not show a statistically significant association with SAH, this finding should be interpreted with caution, as regular physical activity is widely recognized as a fundamental strategy for the prevention and treatment of this condition [21]. Findings from a recent longitudinal study examining PAL and SAH outcomes in Brazil indicated that individuals classified as very physically active, regardless of sex, had a significantly lower risk of developing SAH. Furthermore, women demonstrated an even greater reduction in risk compared with men when engaging in higher volumes and intensities of exercise over time [22].

Meta-analyses demonstrate that both aerobic exercise and resistance training promote clinically relevant reductions in blood pressure, including in postmenopausal women [23,24]. Resistance training, in particular, has gained attention for its beneficial effects on endothelial function, reduction in arterial stiffness, and favorable changes in body composition, including reductions in visceral fat [25,26].

Although alcohol consumption was not associated with SAH in the present study, the high prevalence of participants reporting recreational alcohol use (51.0%) deserves attention. Evidence indicates that no safe level of alcohol consumption exists with respect to cardiovascular risk [27] and that even recreational alcohol intake is closely associated with increased blood pressure through mechanisms such as sympathetic activation, increased oxidative stress, and endothelial dysfunction [28]. Similarly, smoking is a well-established risk factor for cardiovascular diseases, as it exacerbates inflammation, arterial stiffness, and endothelial dysfunction [29]. The absence of statistically significant associations for these variables in the present study may be attributed to the small sample size, which limited statistical power.

Among the limitations of the present study are its cross-sectional design, which precludes causal inferences; the small sample size, although consistent with the proposed study model; and the potential recall bias related to self-reported physical activity level, smoking, and alcohol consumption. In addition, alcohol consumption was assessed in a dichotomous manner without quantification of intake, and detailed information on dietary habits, including sodium intake, was not collected, which may limit a more comprehensive analysis of the determinants of systemic arterial hypertension.

The identification of systemic arterial hypertension was based on self-reported previous medical diagnosis and the regular use of antihypertensive medication, without the performance of standardized blood pressure measurements in a clinical setting by healthcare professionals. Likewise, objective blood pressure monitoring methods, such as Home Blood Pressure Monitoring or Ambulatory Blood Pressure Monitoring, which could provide a more accurate assessment of blood pressure levels, were not used in the present study.

Furthermore, to estimate the PAL, the minutes corresponding to resistance training sessions performed as part of the research program were excluded in the secondary classification of PAL in order to avoid overestimation of habitual physical activity; however, this approach may be subject to potential misclassification.

Additionally, factors related to the etiology of systemic arterial hypertension, such as dietary habits and stress, and, in the case of women, the use or non-use of HRT, should be considered. It is important to highlight that body weight reduction, closely related to body mass index and waist circumference, is multifactorial. Therefore, the results of this study should be interpreted with caution regarding their generalizability, since the sample consisted of physically active women from a single municipality, which limits extrapolation to populations with different sociodemographic and behavioral characteristics. Thus, future studies should consider collecting more detailed data on dietary habits, sodium intake, alcohol consumption, and overall PAL in order to enable a more comprehensive understanding of the determinants of systemic arterial hypertension.

Among the strengths of the present study are the scarcity of research involving postmenopausal women in Brazil, the assessment of potentially important determinants of health outcomes such as PAL, the evaluation of the association between chronic health conditions, and the examination of the impact of sociodemographic factors on health outcomes and potential public health interventions. These findings highlight the importance of promoting a healthy and active lifestyle as a preventive strategy against SAH, taking into account the specific characteristics of the sample and emphasizing the relevance of multidisciplinary approaches across all levels of women’s health care.

## 5. Conclusions

After the analyses and associations, it was possible to conclude that factors such as overweight and obesity, excess central adiposity, and low educational level are associated with risk factors for SAH in the studied sample, with a small to moderate tendency toward association with a diagnosis of DM. These findings reinforce the relevance of health care and underscore the importance of monitoring these and other parameters in the aging process of women, highlighting the importance of a healthy lifestyle even among physically active women and the role of multidisciplinary care in postmenopausal health.

## Figures and Tables

**Table 1:** Sociodemographic, behavioral, and health characteristics of the sample (*n* = 55).

Variables	Values
**Age (years)** (mean/SD)	66.0 ± 4.9
**Weight (kg)** (mean/SD)	68.8 ± 12.5
**Height (m)** (mean/SD)	1.56 ± 0.06
**Postmenopausal duration (years)** (mean/SD)	15.2 ± 5.1
**Ethnicity** (*n*/%)	
Black + Brown	24 (43.6%)
White	31 (56.4%)
**Educational level (years)** (*n*/%)	
0–7	11 (20.0%)
8–11	7 (12.7%)
≥12	37 (67.3%)
**BMI (kg/m^2^)** (mean/SD)	27.95 ± 4.93
**BMI** (*n*/%)	
Underweight + normal weight	28 (50.9%)
Overweight + obesity	27 (49.1%)
**WC (cm)** (*n*/%)	
80–88 (16)	16 (29.1%)
≥88 (39)	39 (70.9%)
**PAL** (*n*/%)	
Physically inactive	16 (29.1%)
Active	28 (50.9%)
Very active	11 (20.0%)
**Smoking status** (*n*/%)	
Yes	8 (14.5%)
No	47 (85.5%)
**Alcohol consumption** (*n*/%)	
Si	28 (51.0%)
No	27 (49.0%)
**DM** (*n*/%)	
Yes	6 (11.0%)
No	49 (89.0%)
**SAH** (*n*/%)	
Yes	24 (43.6%)
No	31 (56.4%)

Data are presented as mean ± standard deviation (m ± SD) or as frequency and percentage (*n*/%). Abbreviations: BMI—body mass index; WC—waist circumference; PAL—physical activity level; DM—diabetes mellitus; SAH—systemic arterial hypertension. Units: kg—kilogram; m—meter; kg/m^2^—kilogram per meter squared; cm—centimeter.

**Table 2:** Association between SAH and sociodemographic, behavioral, and health-related variables (*n* = 55).

Variables	Subcategories (Mean)	SAH		*p*-Value	ES
		Yes (*n*/%)	No (*n*/%)		
BMI	Eutrophic (28)	8 (28.6%)	20 (71.4%)	0.032 *^a^	0.30 ^†^
	Overweight + Obesity (27)	16 (59.3%)	11 (40.7%)		
WC	80–87 cm (16)	2 (12.5%)	14 (87.5%)	0.006 *^a^	0.40 ^†^
	≥88 cm (39)	22 (56.4%)	17 (43.6%)		
PAL	Inactive (44)	20 (45.5%)	24 (54.5%)	0.731 ^b^	0.13 ^†^
	Active (11)	4 (36.4%)	7 (63.6%)		
DM	Presence (6)	5 (83.3%)	1 (16.7%)	0.074 ^b^	0.28 ^†^
	Absence (49)	19 (38.8%)	30 (61.2%)		
Smoking status	Presence (8)	4 (50.0%)	4 (50.0%)	0.717 ^b^	0.16 ^†^
	Absence (47)	20 (42.6%)	27 (57.4%)		
Alcohol consumption	Presence (28)	10 (35.7%)	18 (64.3%)	0.282 ^a^	0.08 ^†^
	Absence (27)	14 (51.9%)	13 (48.1%)		
Educational level	0–7 years (11)	10 (90.9%)	1 (9.1%)	0.003 *^b^	0.47 ^‡^
	8–11 years (7)	3 (42.9%)	4 (57.1%)		
	≥12 years (37)	11 (29.7%)	26 (70.3%)		
Ethnicity	Black + Brown (24)	11 (45.8%)	13 (54.2%)	0.796 ^a^	0.11 ^†^
	White (31)	13 (41.9%)	18 (58.1%)		

Data are presented as frequency and percentage (*n*/%). Abbreviations: BMI—body mass index; WC—waist circumference; PAL—physical activity level; DM—diabetes mellitus; SAH—systemic arterial hypertension; ES—effect size; ^‡^—φ (Phi) was used for 2 × 2 contingency tables; ^†^—Cramér’s V was used for tables larger than 2 × 2.; ^a^—Pearson’s chi-square test; ^b^—Fisher’s exact test. Units: m—meter; cm—centimeter. A significance level of *p* < 0.05 was used to determine statistical significance (*).

**Table 3:** Risk and 95% confidence interval for factors associated with SAH in postmenopausal women (*n* = 55).

Variable	OR Adjusted (ExpB)	95% CI	*p*-Value	Crude OR (ExpB)	95% CI	*p*-Value
**WC (cm)**						
80–88 ^#^	1.00	-	0.055 *	1.00	-	0.007 *
≥88	5.54	0.965–31.872		9.05	1.809–45.370	
**DM**						
Presence	8.18	0.607–110.233	0.113	7.89	0.855–72.879	0.068
Absence ^#^	1.00	-		1.00	-	
**Educational level (** **years)**						
0–7	13.98	1.505–129.833	0.020 *	23.63	2.690–207.667	0.004 *
8–11	1.35	0.165–11.021	0.779	1.77	0.339–9.273	0.498
≥12 ^#^	1.00	-		1.00	-	

Results are presented as odds ratios (OR), estimated using the exponentiated coefficient (ExpB), with their respective 95% confidence intervals (95% CI) and *p*-values. The reference category for each variable was defined as OR = 1.00 (^#^). A significance level of *p* < 0.05 was used to determine statistical significance (*). Abbreviations: WC—waist circumference; DM—diabetes mellitus; 95% CI—95% confidence interval. Units: cm—centimeter.

## Data Availability

The data supporting this study are available from the corresponding author upon reasonable request for non-commercial research purposes.

## Abbreviations

The following abbreviations are used in this manuscript:

SAH	Systemic arterial hypertension
BMI	Body Mass Index
WC	Waist Circumference
PAL	Physical Activity Level
DM	Diabetes Mellitus
